# The small molecule Bcl-2/Mcl-1 inhibitor TW-37 shows single-agent cytotoxicity in neuroblastoma cell lines

**DOI:** 10.1186/s12885-019-5439-1

**Published:** 2019-03-18

**Authors:** Stefanie Klenke, Neval Akdeli, Patrick Stelmach, Lukas Heukamp, Johannes H. Schulte, Hagen S. Bachmann

**Affiliations:** 1 Institute of Pharmacogenetics, University Hospital Essen, Essen, Germany; 2 Department of Anesthesiology and Intensive Care, University Hospital Essen, Essen, Germany; 3 NEO New Oncology AG, Cologne, Germany; 4 Institute of Hematopathology Hamburg, Hamburg, Germany; 5 Department of Pediatric Oncology and Hematology, Charité Berlin, Berlin, Germany; 6 Institute of Pharmacology and Toxicology, Center for Biomedical Education and Research (ZBAF), School of Medicine, Faculty of Health, Witten/Herdecke University, Stockumer Str 10, 58453 Witten, Germany

**Keywords:** Bcl-2, Mcl-1, TW-37, Neuroblastoma, apoptosis

## Abstract

**Background:**

High-risk neuroblastoma with N-Myc amplification remains a therapeutic challenge in paediatric oncology. Antagonism of pro-death Bcl-2 homology (BH) proteins to pro-survival BH members such as Mcl-1 and Bcl-2 has become a treatment approach, but previous studies suggest that a combined inhibition of Bcl-2 and Mcl-1 is necessary. TW-37 inhibits Mcl-1 and Bcl-2 with almost the same affinity. However, single-agent cytotoxicity of TW-37 in neuroblastoma cell lines has not been investigated.

**Methods:**

Cell viability, apoptosis, proliferation and changes in growth properties were determined in SKNAS, IMR-5, SY5Y and Kelly cells after treatment with TW-37. After transfection with Mcl-1 or Bcl-2 siRNA, apoptosis and proliferation were investigated in Kelly cells. Mice with Kelly cell line xenografts were treated with TW-37 and tumor growth, survival and apoptosis were determined.

**Results:**

Cell lines with N-Myc amplification were more sensitive to TW-37 treatment, IC50 values for IMR-5 and Kelly cells being 0.28 μM and 0.22 μM, compared to SY5Y cells and SKNAS cells (IC50 0.96 μM and 0.83 μM). Treatment with TW-37 resulted in increased apoptosis and reduced proliferation rates, especially in IMR5 and Kelly cells. Bcl-2 as well as Mcl-1 knockdown induced apoptosis in Kelly cells. TW-37 led to a decrease in tumor growth and a favorable survival (*p* = 0.0379) in a Kelly neuroblastoma xenografts mouse model.

**Conclusion:**

TW-37 has strong single-agent cytotoxicity in vitro and in vivo. Therefore, combined inhibition of Bcl-2/Mcl-1 by TW-37 in N-Myc amplified neuroblastoma may represent an interesting therapeutic strategy.

**Electronic supplementary material:**

The online version of this article (10.1186/s12885-019-5439-1) contains supplementary material, which is available to authorized users.

## Background

Neuroblastoma is the third most common tumor entity in childhood and is responsible for 15% of cancer deaths in children [1]. Despite development and testing of multiple therapy strategies such as chemotherapy and surgery, high-risk neuroblastoma remains a tumor with a poor prognosis [2]. Risk stratification of neuroblastoma patients is based on diverse prognostic factors, grade of tumor differentiation, N-Myc oncogene amplification, 11q deletion and DNA ploidy. Nowadays, about half of all diagnosed cases are classified as high-risk for disease relapse, while overall survival rates still show only modest improvement, less than 40% at 5 years [3]. Therefore, the future challenge is to develop risk-based therapies to improve outcome [4]. But augmenting treatment efficacy for the high-risk group will likely require the development of additional therapies based on targetable pathways specifically activated in neuroblastomas with N-Myc amplification. One important therapeutic issue is restoring drug sensitivity, because neuroblastoma-derived cells maintain competent mitochondrial apoptotic signalling [5] and are dependent on these pathways for response to various cellular stressors such as N-Myc overexpression or cytotoxic agents [6, 7]. Entry into the common pathway of mitochondrial apoptosis is governed by competitive binding of pro-death Bcl-2 homology (BH) proteins to pro-survival BH members such as Mcl-1 and Bcl-2, which neutralize activation of pro-death Bak and/or Bax. Chemoresistance may derive from the activation of pro-survival BH proteins, which tip the cellular balance away from apoptosis [8, 9].

The expression of Mcl-1 and Bcl-2 correlated to clinical prognostic factors and survival in neuroblastoma patients [10]. Therefore, BH antagonism has become a treatment approach in neuroblastoma, and several agents have been developed with different selectivity to inhibit Bcl-2 and Mcl-1. ABT-737, that binds with subnanomolar affinity to Bcl-2, Bcl-W and Bcl-xL, but has no appreciable affinity for Mcl-1, has shown to induce cell death in neuroblastomas [10, 11]. However, resistance to ABT-737 has been reported due to constitutive upregulation of Mcl-1, and drug activity was restored if Mcl-1 was simultaneously antagonized [10]. AT-101, which also neutralizes Mcl-1, was more active against neuroblastoma cells, but concomitant Mcl-1 knockdown further increased potency, suggesting that Mcl-1 antagonism was incomplete [10]. These results lead to the conclusion, that the combined inhibition of Bcl-2 and Mcl-1 may be a useful therapeutic strategy in the treatment of neuroblastoma. This assumption is confirmed by a recent study, which demonstrated that neuroblastoma cells might survive ABT-199 treatment, a specific Bcl-2 inhibitor, due to acute upregulation of Mcl-1. In-vitro inhibition of Mcl-1 sensitized neuroblastoma cell lines to ABT-199 [12]. Another study demonstrated that N-Myc amplified neuroblastomas were sensitive to ABT-199 [13]. Sensitivity occurred in part through low anti-apoptotic Bcl-xL expression, and upregulation of the Mcl-1 inhibitor NOXA. However, N-Myc amplified neuroblastomas could be further sensitized to ABT-199 with the Aurora Kinase A inhibitor MLN8237, which results in a downregulation of Mcl-1 [13].

Therefore, there is ongoing research to identify an inhibitor of Bcl-2 and Mcl-1 in N-Myc amplified neuroblastomas. An interesting lead candidate is TW-37, a second-generation benzenesulfonyl derivative of gossypol [14], which inhibits Mcl-1 and Bcl-2 with almost the same affinity (Ki of 260 nmol/L and 290 nmol/L) and which has also low affinity to Bcl-xL [14, 15]. TW-37 binds to the BCL-2 homology domain 3 (BH3) groove of Bcl-2 preventing the heterodimerization of proapoptotic proteins with Bcl-2 and subsequently allowing them to induce apoptosis [15]. Recent studies indicate that TW-37 is able to inhibit the growth of a broad range of cancer cells, since it induces S-phase cell cycle arrest with regulation of several important cell cycle related genes, including p27, p57, E2F-1, cdc25A, CDK4, cyclin A, cyclin D1 and cyclin E [16, 17]. However, single-agent cytotoxicity of TW-37 in neuroblastoma cell lines has not been investigated.

Therefore, we explored in this study the effects of TW-37 on apoptosis and proliferation rate in neuroblastoma cell lines and in an in vivo xenograft mouse model with special regard to N-Myc amplified neuroblastoma cell lines.

## Methods

### Cell lines and TW-37 stock solution

The human neuroblastoma cell lines, SKNAS, IMR-5, SY5Y and Kelly, were grown in RPMI 1640 (Fisher Scientific, Schwerte, Germany) supplemented with 10% standardized fetal bovine serum (Merck Millipore, Darmstadt, Germany) and 1% Penicillin/Streptomycin (GE Healthcare, Freiburg, Germany). All cell lines were obtained in 2013. SY5Y (CRL-2266) and SKNAS (CRL-2137) cell lines were obtained from the American Tissue Culture Collection (ATCC), Kelly (ACC355) were purchased from the DSMZ-German Collection of Microorganism and Cell Cultures (Germany) and the neuroblastoma cell line IMR-5, a subclone of IMR-32 (CCL-127), was kindly provided by Dr. Alexander Schramm (Department of Molecular Oncology, West German Cancer Centre, Essen, Germany). The identities of the four cell lines were verified by STR genotyping performed by Eurofins Medigenomix (Ebersberg, Germany). All cell lines were repeatedly tested in our laboratory for mycoplasma contamination, which could be excluded.

Amplification of N-Myc has been observed in IMR-5 and Kelly cells, while SY5Y and SKNAS are negative for amplification of N-Myc [18]. TW-37 (Selleck Chemicals, Munich, Germany) was dissolved in DMSO and stored as a 100 mM stock solution at − 20 °C until use.

### Protein extraction and Western blot analysis

To determine protein expression of Bcl-2 and Mcl-1, untreated cell lines were cultured for 72 h before whole cell lysates were extracted. In small interfering RNA (siRNA) knockdown experiments, cells were grown for 72 h after transfection before whole cell lysates were extracted. Cells were washed with PBS, suspended in ice cold RIPA buffer (Tris-HCL, 50 mM, pH 7.4; Np-40, 1%; Desoxycholic acid sodium salt, 0,25%; NaCl, 150 mM; EDTA, 1 mM, complete protease inhibitor (Roche, Mannheim, Germany)) and shaken for 15 min at 4 °C. The lysate was centrifuged at 1300 rpm for 15 min at 4 °C. The supernatant was harvested. Protein concentration was determined with Pierce™ BCA Protein Assay Kit (Fisher Scientific, Schwerte, Germany) following manufacturer’s instructions. The protein lysates were snap-frozen in liquid nitrogen and stored at − 80 °C until use. Before western blotting proteins were mixed with Laemmli buffer and were denatured 5 min at 95 °C. After electrophoresis, the gels were transferred to polyvinylidene difluoride membranes. Transfer membranes were incubated at 4 °C overnight using the following antibodies: Bcl-2 (sc-783, Santa Cruz Biotechnology, Heidelberg, Germany); Mcl-1 (sc-12,756, Santa Cruz Biotechnology, Heidelberg, Germany); ß-Actin (Merck Millipore, Darmstadt, Germany).

### Cell proliferation, viability, and cell cycle analysis

Human neuroblastoma cell lines were seeded onto 96-well plates (2 × 10^4^ cells per well). After 24 h of incubation, cells were treated with variable concentrations of TW-37, range: 0.01–10 μM. The cells were incubated for 48 h, the RPMI medium was replaced daily and TW-37 concentrations were kept constant during the experiment. Subsequently cell viability was measured using the 3-(4,5-dimethylthiazol-2-yl)-2,5-diphenyltetrazolium bromide (MTT)-Assay (Roche, Mannheim, Germany), following manufacturer’s protocol. For cell cycle analysis, cell lines were treated with 1 μM TW-37. After 48 h of growth, cells were trypsinized, washed with PBS and incubated with propidium iodide for 15 min to stain DNA. The DNA content was analyzed by Cytomics FC500 flow cytometer (Beckmann Coulter). For measurement of apoptosis and proliferation enzyme-linked immunosorbent assay (ELISA) was performed following the manufacturer’s instructions (Cell Death Detection ELISA, Roche, Mannheim, Germany and BrdU ELISA, Roche, Mannheim, Germany). Cell Death ELISA and BdrU ELISA as described above were also performed on Kelly cells treated with siRNA. Therefore, Kelly cells were plated onto a 12-well plate and transiently transfected (HiPerFect transfection reagent Qiagen, Hilden, Germany) with either siRNA directed against Bcl-2 (Qiagen, Hilden, Germany) or Mcl-1 (Qiagen, Hilden, Germany) following manufacturer’s protocol. In addition, untreated cells and cells with mock transfection were cultured. After 72 h of transfection, apoptosis and proliferation was measured by ELISA as described above.

### Kelly cell xenograft tumor in nude mice

8 week old female athymic NCR (nu/nu) mice were randomized into 2 groups. A TW-37 and a vehicle control group (*n* = 4 mice per group). In order to establish Kelly cell xenograft tumor, Kelly cells were allowed to grow up to a confluence of 80%. Then the cells were washed with PBS and suspended in 200 μl Matrigel (BD Bioscience, Heidelberg, Germany). After that 2 × 10^7^ cells per mouse, *n* = 8 were injected s.c. in the flank. On days 5–7 and 12–14 mice were treated with either TW-37 or vehicle control. Thirty minutes before TW-37 treatment, the drug was dissolved in 18:1:1 *v*/v PBS/Tween 80/ethanol. Mice were treated by tail vein injection of 20 mg/kg body weight TW-37 in 300 μl 18:1:1 v/v PBS/Tween 80/ethanol. The tumor volume was determined by digital calliper 3 times a week. Upon reaching a tumor volume of more than 1000 mm^3^ mice were euthanized by cervical dislocation. The tumor was removed, formalin fixed and paraffin embedded. The animal experiments were performed in accordance with the Council of Europe guidelines for accommodation and care of laboratory animals and protocols were approved by the North Rhine-Westphalia State Agency for Nature, Environment and Consumer Protection.

### Immunohistochemistry

Xenograft tumors of mice treated with either TW-37 or control were paraffin embedded. All tumors were clinically and pathologically identified as being the primary and only neoplastic lesion. Briefly, 3-μm-thick sections of formalin-fixed paraffin-embedded (FFPE) tumors were deparaffinized, and antigen retrieval was performed by boiling the section in citrate buffer at pH 6 or EDTA at pH 9 for 20 min. As primary antibody Ki67 (mib-1, 1:100, pH 6, Thermo Scientific, Waltham, MA, USA) was used. Corresponding secondary antibody detection kits for reduced background on murine tissue were used (Histofine Simple Stain Mouse MAX PO, medac) and stained on an automated stainer (LabVision Autostainer 480S, Thermo Scientific). For cleaved caspase 3 (Cell Signaling Technologies, Danvers, MA, USA) staining of paraffin sections, antigens were retrieved with EDTA buffer (1 mmol/L EDTA, pH 8.0), peroxidases blocked 10 min in 3% hydrogen peroxide, and the antibodies were diluted in Tris-buffered saline containing 1% bovine serum albumin and 5% normal goat serum 1:200. The histochemistry was performed with Super Sensitive Link Label IHC Detection system (BioGenex, San Ramon, CA, USA) and visualized with diaminobenzidine (DAB; Dako, Giostrup, Denmark).

### Statistics

Student’s t-test was used for comparison of treatment groups. Half maximal inhibitory concentrations (IC50) were calculated from nonlinear regression (curve fit) and Kaplan Meier survival analysis with log rank test were used to analyze survival of the mouse cohorts. Graphs are mean +/− SD. All analyses were performed using SPSS 20 (IBM, Armonk, NY, USA) and Graph Pad Prism 7.0 (GraphPad Software, LaJolla, CA, USA). Differences with *p*-values < 0.05 were considered significant and all p-values are two-tailed.

## Results

### TW-37 reduces viability of neuroblastoma cell lines, with strongest effect in cell lines with N-Myc amplification

**Fig. 1:**
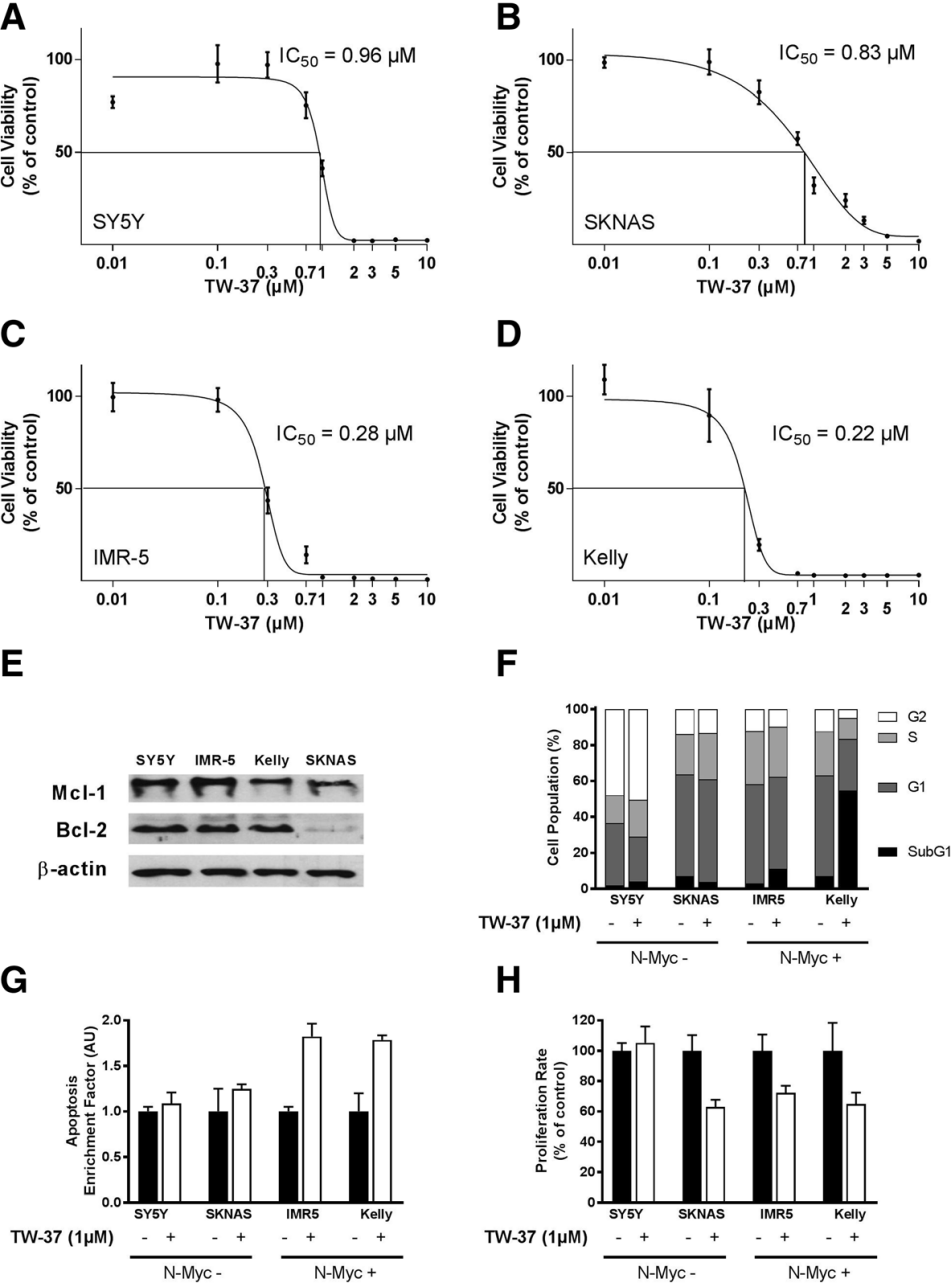
Cell viability, measured in MTT-assay in Kelly (**a**), IMR-5 (**b**), SKNAS (**c**) and SY5Y (**d**) cells 72 h after treatment with variable concentrations of TW-37. The IC-50 value was determined for each cell line. **e** Western Blot of whole cell lysate of four neuroblastoma cell lines with antibodies against Bcl-2 and Mcl-1 protein, and the housekeeping protein β-actin. **f** SKNAS, SY5Y, IMR5 and Kelly cells were treated with 1 μM TW-37 following cell cycle analysis by FACS. Diagrammed is the percentage of cells in the different cell cycles. **g** Apoptosis was measured in SKNAS, SY5Y, IMR5 and Kelly cells after treatment with 1 μM TW-37. The enrichment factor was used as a parameter of apoptosis. **h** Proliferation SKNAS, SY5Y, IMR5 and Kelly cells after treatment with 1 μM TW-37 was measured by ELISA. The proliferation rate is given as a percentage of control

In order to evaluate the effect of the small-molecular inhibitor TW-37, cell lines were treated with variable concentrations of TW-37 in vitro*.* In all cell lines, a significant decrease in cell viability was detected by MTT-assay. In SY5Y cells the IC50 value was achieved at 0.96 μM (Fig. 1a) in SKNAS cells at 0.83 μM (Fig. 1b), in IMR-5 cells at 0.28 μM (Fig. 1c) and in Kelly cells at 0.22 μM (Fig. 1d). Cells lines with an N-Myc amplification (IMR-5 and Kelly cells) were more sensitive to TW-37 treatment indicating by clearly lower IC-50 values than cells lines without an N-Myc amplification (SY5Y and SKNAS cells).

Protein expression analysis in untreated cell lines revealed expression of both, Bcl-2 and Mcl-1. However, SKNAS cells expressed Bcl-2 to a much lesser extent than the other cell lines (Fig. 1e).

When the cells were treated with 1 μM TW-37, in fluorescence-activated cell sorting (FACS) analysis the fraction of apoptotic cells, reported by the higher percentage of sub-G1 cells was increased in cells lines with N-Myc amplification. The strongest effect was observed in Kelly cells. In cells without N-Myc amplification, there was no clear difference in apoptosis between TW-37 treated and non-treated cells (Fig. 1f). A cell death ELISA revealed a significantly higher fraction of apoptotic cells in IMR5 and Kelly cells and only a marginal effect in SY5Y and SKNAS cells after treatment with 1 μm TW-37 (Fig. 1g), confirming results of FACS analysis. In a cell proliferation ELISA a clear inhibition of proliferation in SKNAS, IMR5 and Kelly cells after treatment of 1 μM TW-37 was observed, but no effect was seen in SY5Y cells (Fig. 1h).

**Fig. 2:**
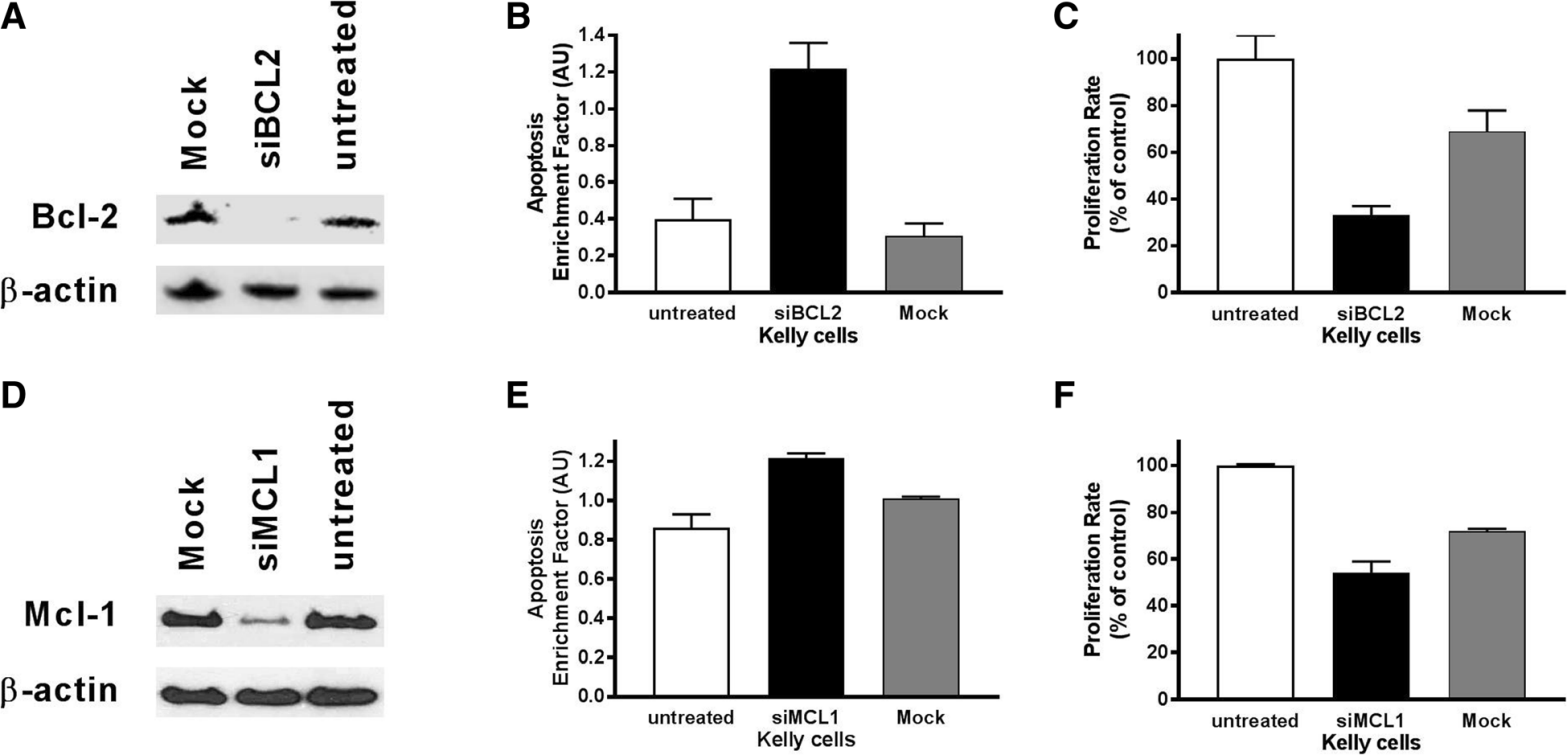
**a** Western Blot of whole cell lysate after transfection with Bcl-2 siRNA and with antibodies against Bcl-2, and the housekeeping protein β-actin. **b** Apoptosis was measured in Kelly cells after transfection with Bcl-2 siRNA. The enrichment factor was used as a parameter of apoptosis. **c** Proliferation was measured in Kelly cells after transfection with Bcl-2 siRNA. The proliferation rate is given as a percentage of control. **d** Western Blot of whole cell lysate after transfection with Mcl-1 siRNA and with antibodies against Mcl-1, and the housekeeping protein β-actin. **e** Apoptosis was measured in Kelly cells after transfection with Mcl-1 siRNA. The enrichment factor was used as a parameter of apoptosis. **f** Proliferation was measured in Kelly cells after transfection with Mcl-1 siRNA. The proliferation rate is given as a percentage of control

A selective knockdown with siRNA against Bcl-2 and Mcl-1 was performed in Kelly cells (Fig. 2a and d), since this cell line showed strongest effect on treatment with TW-37 in previous experiments. Indeed, the siRNA mediated knockdown of Bcl-2 as well as of Mcl-1 mimicked the effect of TW-37 treatment: an increase in apoptosis (Fig. 2b and e), and an inhibition of proliferation were observed (Fig. 2c and f), whereas the mock transfection did not or only to a lesser extent affect proliferation and apoptosis. These in vitro results provide strong evidence for the impact of TW-37 on cell viability and proliferation in neuroblastoma cell lines.

### In a xenograft model TW-37 revealed a significant anti-tumor effect

**Fig. 3:**
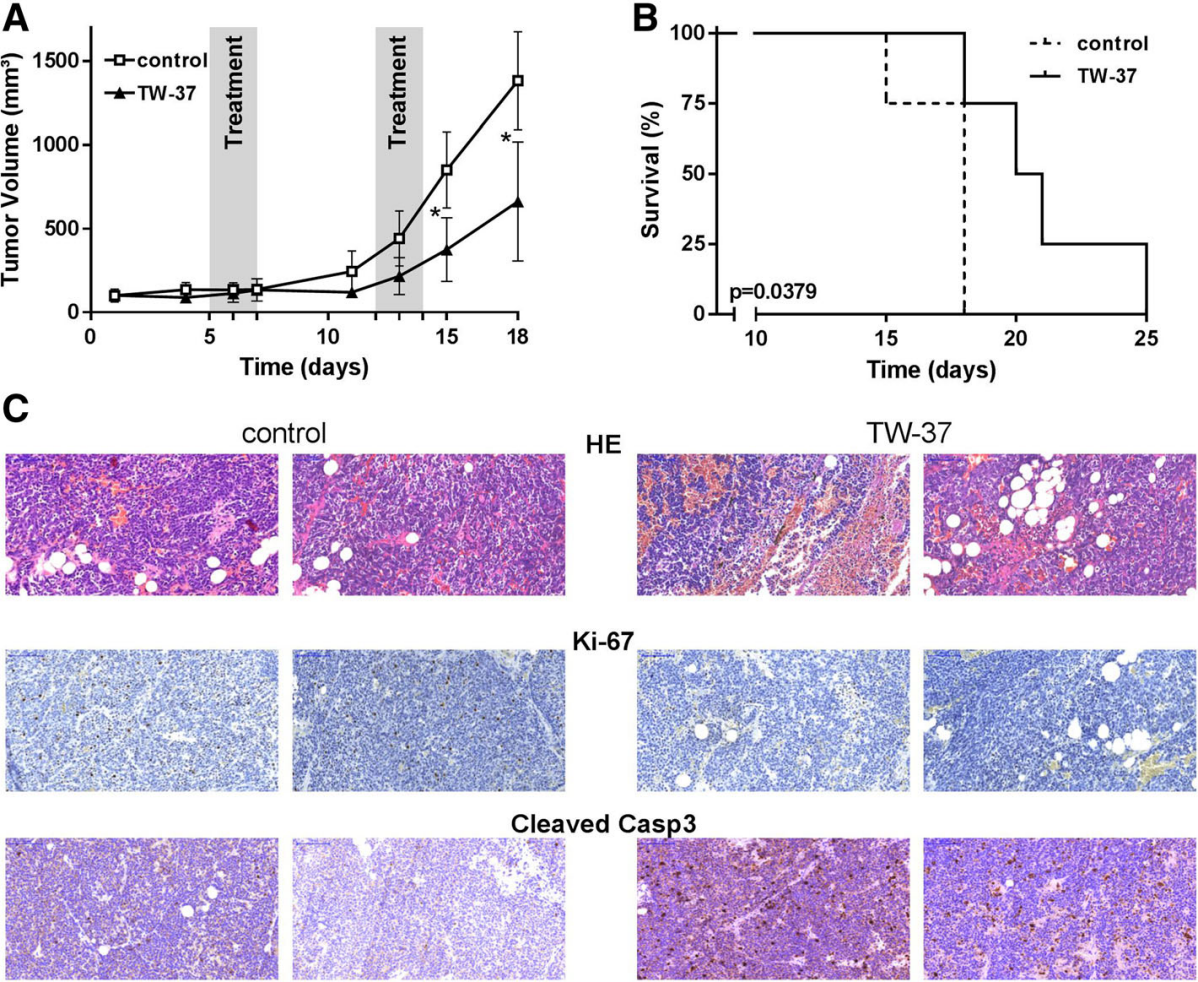
**a** After establishing Kelly cell xenograft tumor, mice were treated with either TW-37 (*n* = 4) or control (*n* = 4) on days 5–7 and 12–14. The tumor volume was determined 3 times a week. **b** Kaplan Meier survival analysis for mice treated with TW-37 (*n* = 4) compared with controls (*n* = 4). **c** HE and Immunohistochemistry with Ki-67 and Cleaved Caspase 3 of kelly cell xenograft tumor of mice after treatment of TW-37 vs. control. Magnification scale bar 100 μm

To evaluate the effects of TW-37 in vivo, mice with a neuroblastoma Kelly cell xenograft were treated with TW-37 by tail vein injection. The treatment was well tolerated and no serious side effects were observed. There was no reduction in tumor volume after initial treatment with TW-37 in nude mice with existing Kelly cell xenograft, but at 15 days (*p* = 0.0103) and 18 days (*p* = 0.0364, Fig. 3a). Considering a primary end point of > 1000 mm^3^ tumor volume, a significantly longer survival of TW-37 treated mice was observed in comparison to mice treated with placebo (Fig. 3b). The evaluation of the xenograft tumor after TW-37 treatment revealed in an immunohistochemical analysis an increase in apoptotic cells, indicated by an increase in caspase 3 (Fig. 3c, high resolution Additional files 1, 2, 3, 4, 5, 6, 7 and 8). Furthermore, a decrease in proliferation rate was observed indicated by a decrease in Ki-67 (Fig. 3c, high resolution Additional files 9, 10, 11 and 12). These findings are in line with our previous findings in in vitro experiments. Thus, the treatment with TW-37 in mice with Kelly-cell xenograft tumor revealed that TW-37 has also in vivo a clear effect on apoptosis leading to a delay in tumor growth.

## Discussion

Despite development and testing of multiple therapy strategies such as chemotherapy and surgery, high-risk neuroblastoma remains a tumor with a poor prognosis [2]. To increase treatment efficacy in the high-risk group, inhibitors of Bcl-2 and Mcl-1 have been developed to restore drug sensitivity, however, previous studies lead to the conclusion that a combined inhibition of Bcl-2 and Mcl-1 may be necessary. Therefore, we evaluated the effect of TW-37, which inhibits Mcl-1 and Bcl-2 with almost the same affinity and which has also low affinity to Bcl-xL [14, 15] in neuroblastoma cell lines.

In this study, we can demonstrate that TW-37 has strong single-agent cytotoxicity in vitro and in vivo in N-Myc amplified neuroblastoma. Treatment with TW-37 results in reduced apoptosis, proliferation, and cell viability in the N-Myc amplified neuroblastoma cell lines (IMR-5 and Kelly cells) and in a decrease in tumor growth and a favorable survival accompanied with an increase in apoptotic cells and reduced proliferation in mice with Kelly cell xenograft.

N-Myc amplification in neuroblastoma is a poor prognostic factor [19] and in a recent study it could be demonstrated that only N-Myc amplified cell lines showed sensitivity to ABT-199, compared with N-Myc wildtype cell lines [13]. Further analysis demonstrated that the Mcl-1 inhibitor NOXA, encoded by PMAIP1, was significantly higher in N-Myc amplified neuroblastomas suggesting that increased NOXA expression was a contributing factor to ABT-199 sensitivity observed in N-Myc amplified neuroblastoma cells.

Therefore, a clear line must be drawn between N-Myc amplified neuroblastoma cell lines compared to N-Myc wild type neuroblastoma cell lines due to differential targetable pathways. This assumption is confirmed by our results that we observed a stronger effect in apoptosis, proliferation, and cell viability after treatment with TW-37 in the N-Myc amplified neuroblastoma cell lines (IMR-5 and Kelly cells) compared to N-Myc wild type neuroblastoma cell lines (SY5Y and SKNAS cells). Therefore, combined Bcl-2/Mcl-1 inhibition should be further elucidated as a treatment option in neuroblastoma with N-Myc amplification. Neuroblastoma with N-Myc amplification represent the high-risk group [19] and development of additional therapies is warranted due to poor prognosis.

TW-37 is a second-generation benzenesulfonyl derivative of gossypol [14]. Whereas gossypol primary inhibits only Bcl-2, the small-molecular inhibitor TW-37 inhibits Bcl-2 and Mcl-1 with almost the same affinity with Ki values of 0.29 μM, 0.26 μM. To this, TW-37 has also affinity and selectivity for Bcl-xL with Ki values of 1.11 μM [15].

In experiments with siRNA, downregulation of Bcl-2 and Mcl-1 lead to apoptosis and reduced proliferation, as observed with TW-37 treatment, which emphasizes that effects are really a result of the abolished Bcl-2 and Mcl-1 function. However, Ham et al. demonstrated that N-Myc amplified neuroblastomas were sensitive to the Bcl-2 inhibitor ABT-199 and could be further sensitized to ABT-199 with the Aurora Kinase A inhibitor MLN8237, which results in a downregulation of Mcl-1 [13]. Interestingly, sensitivity occurred in part through low anti-apoptotic Bcl-xL expression [13]. Therefore, the fact that TW-37 has also affinity and selectivity to Bcl-xL, might contribute to the observed effect in apoptosis and proliferation after TW-37 treatment.

In the treatment of mice with Kelly cell xenograft with TW-37, a decrease in tumor growth and a favorable survival was observed. Immunohistochemistry of the xenograft tumor revealed that treatment with TW-37 leads to an increase in apoptotic cells and reduced proliferation, suggesting that TW-37 has also significant single-agent activity in vivo*.* However, there was no reduction in tumor volume after initial treatment with TW-37 in mice with existing Kelly cell xenograft.

There have been published only few studies, which investigated effects of TW-37 in vitro and in vivo. In various tumor cell models like, B-cell lymphoma [15], head and neck tumor angiogenesis [17], ovarian cancer [20], and pancreatic cancer [16] treatment with TW-37 was effective alone and especially in combination with chemotherapy. In vivo, TW-37 inhibited tumor angiogenesis and induced tumor apoptosis without significant systemic toxicities. However, TW-37 was given for 10 consecutive days [17]. Combination of TW-37 and cisplatin enhanced the time to tumor as compared to either drug given separately [17]. In a study investigating the effect of TW-37 in combination with radiotherapy on tumor angiogenesis in vivo, TW-37 potentiates the anti-tumor effects of radiotherapy in xenograft of primary human dermal microvascular endothelial cells and human squamous cell carcinoma cells [21]. TW-37 was also applicated for 7–10 consecutive days [21]. From these points, lack of decrease in tumor volume after first TW-37 treatment does not attenuate the observed effect of TW-37. Rather the fact, that we demonstrated an increase in survival by TW-37 treatment even without combination of chemotherapy underlines the efficacy of TW-37. To this, we could demonstrate for the first time an effect of TW-37 in neuroblastoma cell lines. Therefore, in a next step, experiments should be done, for example with primary tumor cells of patients.

Furthermore, in future studies investigation of possible synergistic cytotoxic effect with other chemotherapeutic agents in N-Myc amplified neuroblastoma would be a really interesting, reasonable and promising approach. In a study investigating head and neck cancer, combination of TW-37 and cisplatin showed enhanced cytotoxic effects for endothelial cells and head and neck squamous cell carcinoma as compared with single drug treatment, while TW-37 was more cytotoxic on an equimolar basis than cisplatin [17]. Interestingly, a recent study in nasopharyngeal carcinoma demonstrated that TW-37 promotes apoptosis in nasopharyngeal carcinoma cells under chemotherapeutics treatments but not in nasopharyngeal epithelial cells [22]. Here, TW-37 increased chemosensitivity of nasopharyngeal carcinoma but had no marked influence on normal tissues in mice. Therefore, it would be interesting to investigate in future studies, whether these observations also apply to other cell lines and other tumor entities, such as neuroblastoma cell lines.

To this, in further studies the dosage which is clinically relevant but also well tolerated has to be evaluated. In our study, mice were treated by tail vein injection with 20 mg/kg on days 5–7 and 12–14 and tolerated this treatment well. In 2008, Al-Katib et al. explored the maximum tolerated dose of TW-37 in SCID mice. Animals given 120 mg/kg as intravenous injections (40 mg/kg daily × 3 doses) experienced weight loss of < 5% and had scruffy fur, but showed full recovery 48–72 h after completion of treatment [23]. In a study about TW-37 and nasopharyngeal tumor, mice received 15 mg/kg daily by intraperitoneal injection for 10 days [22]. In this study, TW-37 had no influence on the weight of whole body and key organs, even in chemotherapeutics-treated mice [22]. A further study investigated effects of TW-37 in colorectal cancer [24]. Here, mice were treated via intravenous injection with 10 mg/kg body weight. TW-37 inhibits tumor growth and apparent toxicities among the tested animals were not detected [24]. Therefore, all studies published so far demonstrate that TW-37 is tolerable and effective in mice. However, optimal dosing depends also on the mode of application (intravenous vs. intraperitoneal) and the time and repetitions of injection. Therefore, further studies have to be performed to get reliable information about the concentration/dosage that is clinically relevant. In our model, a dosage of 20 mg/kg body weight TW-37 given intravenously on days 5–7 and 12–14 was well tolerated and led to a decrease in tumor growth and a favorable survival.

## Conclusion

In conclusion, we were able to demonstrate, that TW-37 has strong single-agent cytotoxicity in vitro and in vivo. Therefore, combined inhibition of Bcl-2/Mcl-1 e.g. by TW-37 in N-Myc amplified neuroblastoma may represent an interesting therapeutic strategy.

## Additional files

Additional file 1: Fig. 3c Control1 HE.sss. (BMP 2830 kb)

Additional file 2: Fig. 3c Control2 HE. (BMP 2830 kb)

Additional file 3: Fig. 3c TW37–1 HE. (BMP 2830 kb)

Additional file 4: Fig. 3c TW37–2 HE. (BMP 2830 kb)

Additional file 5: Fig. 3c Control1 Ki-67. (BMP 2830 kb)

Additional file 6: Fig. 3c Control2 Ki-67. (BMP 2830 kb)

Additional file 7: Fig. 3c TW37–1 Ki-67. (BMP 2830 kb)

Additional file 8: Fig. 3c TW37–2 Ki-67. (BMP 2830 kb)

Additional file 9: Fig. 3c Control1 Casp3. (BMP 2830 kb)

Additional file 10: Fig. 3c Control2 Casp3. (BMP 2830 kb)

Additional file 11: Fig. 3c TW37–1 Casp3. (BMP 2830 kb)

Additional file 12: Fig. 3c TW37–2 Casp3. (BMP 2830 kb)

## Abbreviations

BAK: BCL2 antagonist/killer
Bax: BCL2 associated X
Bcl-2: B-cell lymphoma 2
Bcl-w: Bcl-2-like protein 2
Bcl-xL: B-cell lymphoma-extra large
BH: Bcl-2 homology
ELISA: Enzyme-linked immunosorbent assay
FACS: Fluorescence-activated cell sorting
IC50: half maximal inhibitory concentration
Mcl-1: Induced myeloid leukemia cell differentiation protein
NOXA: Phorbol-12-myristate-13-acetate-induced protein 1
siRNA: Small interfering RNA

## References

[1] Maris JM, Matthay KK (1999). Molecular biology of neuroblastoma. J Clin Oncol.

[2] Matthay KK, George RE, Yu AL (2012). Promising therapeutic targets in neuroblastoma. Clin Cancer Res.

[3] Maris JM, Hogarty MD, Bagatell R, Cohn SL (2007). Neuroblastoma. Lancet.

[4] Cheung NK, Dyer MA (2013). Neuroblastoma: developmental biology, cancer genomics and immunotherapy. Nat Rev Cancer.

[5] Goldsmith KC, Hogarty MD (2005). Targeting programmed cell death pathways with experimental therapeutics: opportunities in high-risk neuroblastoma. Cancer Lett.

[6] Paffhausen T, Schwab M, Westermann F (2007). Targeted MYCN expression affects cytotoxic potential of chemotherapeutic drugs in neuroblastoma cells. Cancer Lett.

[7] Ushmorov A, Hogarty MD, Liu X, Knauss H, Debatin KM, Beltinger C (2008). N-myc augments death and attenuates protective effects of Bcl-2 in trophically stressed neuroblastoma cells. Oncogene.

[8] Reed JC (1995). Regulation of apoptosis by bcl-2 family proteins and its role in cancer and chemoresistance. Curr Opin Oncol.

[9] Letai AG (2008). Diagnosing and exploiting cancer's addiction to blocks in apoptosis. Nat Rev Cancer.

[10] Lestini BJ, Goldsmith KC, Fluchel MN, Liu X, Chen NL, Goyal B, Pawel BR, Hogarty MD (2009). Mcl1 downregulation sensitizes neuroblastoma to cytotoxic chemotherapy and small molecule Bcl2-family antagonists. Cancer Biol Ther.

[11] Goldsmith KC, Gross M, Peirce S, Luyindula D, Liu X, Vu A, Sliozberg M, Guo R, Zhao H, Reynolds CP (2012). Mitochondrial Bcl-2 family dynamics define therapy response and resistance in neuroblastoma. Cancer Res.

[12] Bate-Eya LT, den Hartog IJ, van der Ploeg I, Schild L, Koster J, Santo EE, Westerhout EM, Versteeg R, Caron HN, Molenaar JJ (2016). High efficacy of the BCL-2 inhibitor ABT199 (venetoclax) in BCL-2 high-expressing neuroblastoma cell lines and xenografts and rational for combination with MCL-1 inhibition. Oncotarget.

[13] Ham J, Costa C, Sano R, Lochmann TL, Sennott EM, Patel NU, Dastur A, Gomez-Caraballo M, Krytska K, Hata AN (2016). Exploitation of the apoptosis-primed state of MYCN-amplified neuroblastoma to develop a potent and specific targeted therapy combination. Cancer Cell.

[14] Wang G, Nikolovska-Coleska Z, Yang CY, Wang R, Tang G, Guo J, Shangary S, Qiu S, Gao W, Yang D (2006). Structure-based design of potent small-molecule inhibitors of anti-apoptotic Bcl-2 proteins. J Med Chem.

[15] Mohammad RM, Goustin AS, Aboukameel A, Chen B, Banerjee S, Wang G, Nikolovska-Coleska Z, Wang S, Al-Katib A (2007). Preclinical studies of TW-37, a new nonpeptidic small-molecule inhibitor of Bcl-2, in diffuse large cell lymphoma xenograft model reveal drug action on both Bcl-2 and mcl-1. Clin Cancer Res.

[16] Wang Z, Azmi AS, Ahmad A, Banerjee S, Wang S, Sarkar FH, Mohammad RM (2009). TW-37, a small-molecule inhibitor of Bcl-2, inhibits cell growth and induces apoptosis in pancreatic cancer: involvement of Notch-1 signaling pathway. Cancer Res.

[17] Ashimori N, Zeitlin BD, Zhang Z, Warner K, Turkienicz IM, Spalding AC, Teknos TN, Wang S, Nor JE (2009). TW-37, a small-molecule inhibitor of Bcl-2, mediates S-phase cell cycle arrest and suppresses head and neck tumor angiogenesis. Mol Cancer Ther.

[18] Schwab M, Alitalo K, Klempnauer KH, Varmus HE, Bishop JM, Gilbert F, Brodeur G, Goldstein M, Trent J (1983). Amplified DNA with limited homology to myc cellular oncogene is shared by human neuroblastoma cell lines and a neuroblastoma tumour. Nature.

[19] Huang M, Weiss WA (2013). Neuroblastoma and MYCN. Cold Spring Harb Perspect Med.

[20] Wang H, Zhang Z, Wei X, Dai R (2015). Small-molecule inhibitor of Bcl-2 (TW-37) suppresses growth and enhances cisplatin-induced apoptosis in ovarian cancer cells. J Ovarian Res.

[21] Zeitlin BD, Spalding AC, Campos MS, Ashimori N, Dong Z, Wang S, Lawrence TS, Nor JE (2010). Metronomic small molecule inhibitor of Bcl-2 (TW-37) is antiangiogenic and potentiates the antitumor effect of ionizing radiation. Int J Radiat Oncol Biol Phys.

[22] Lu Y, Huang H, Yang H, Chen D, Wu S, Jiang Z, Wang R. Small molecule inhibitor TW-37 is tolerable and synergistic with chemotherapy in nasopharyngeal carcinoma. Cell Cycle. 2017;16(14):1376–83. 10.1080/15384101.2017.1329066 PMC5539829 28696828

[23] Al-Katib AM, Sun Y, Goustin AS, Azmi AS, Chen B, Aboukameel A, Mohammad RM (2009). SMI of Bcl-2 TW-37 is active across a spectrum of B-cell tumors irrespective of their proliferative and differentiation status. J Hematol Oncol.

[24] Lei S, Ding Y, Fu Y, Wu S, Xie X, Wang C, Liang H (2017). The preclinical analysis of TW-37 as a potential anti-colorectal cancer cell agent. PLoS One.

