# Supplementary figures and images for: The small molecule Bcl-2/Mcl-1 inhibitor TW-37 shows single-agent cytotoxicity in neuroblastoma cell lines
Source: BMC Cancer. 2019 Mar 18;19:243. doi: 10.1186/s12885-019-5439-1 (PMC6423774; doi:10.1186/s12885-019-5439-1)

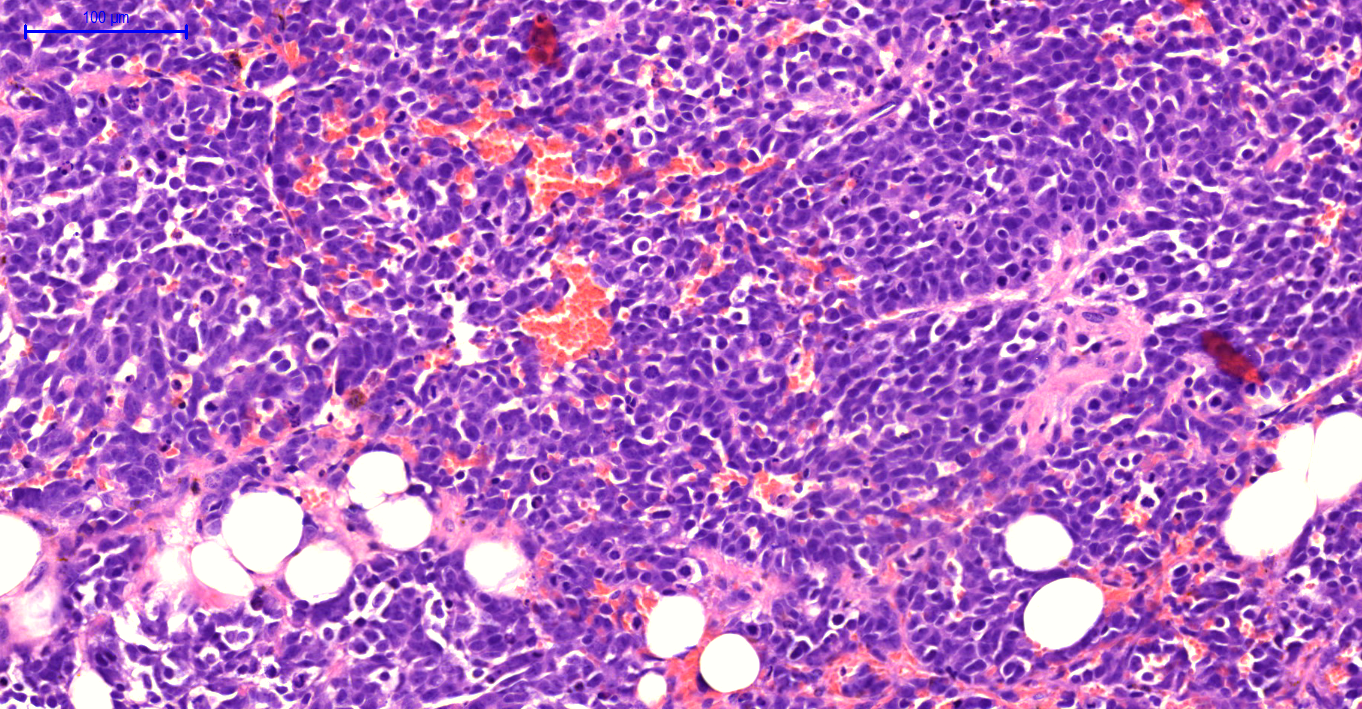

Supplement: Supplementary file 1 — Fig. 3c Control1 HE.sss. (BMP 2830 kb) [file 12885_2019_5439_MOESM1_ESM.bmp]

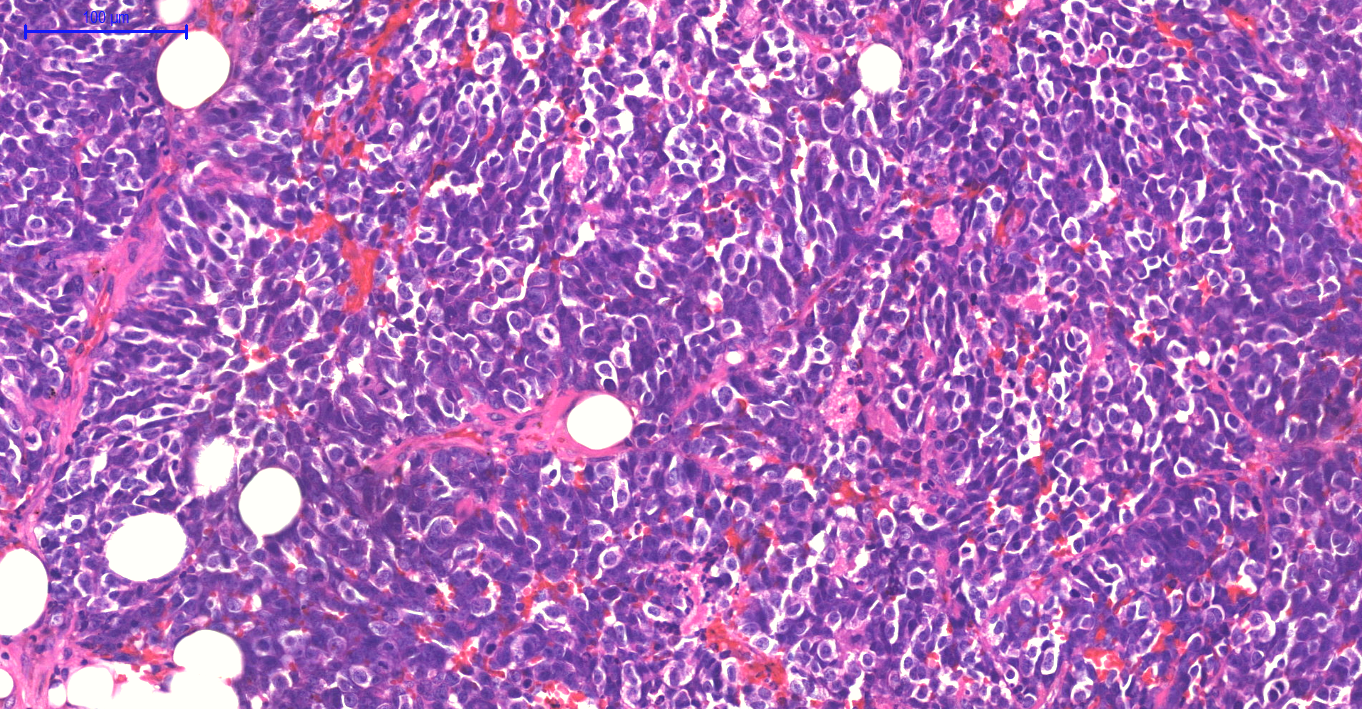

Supplement: Supplementary file 2 — Fig. 3c Control2 HE. (BMP 2830 kb) [file 12885_2019_5439_MOESM2_ESM.bmp]

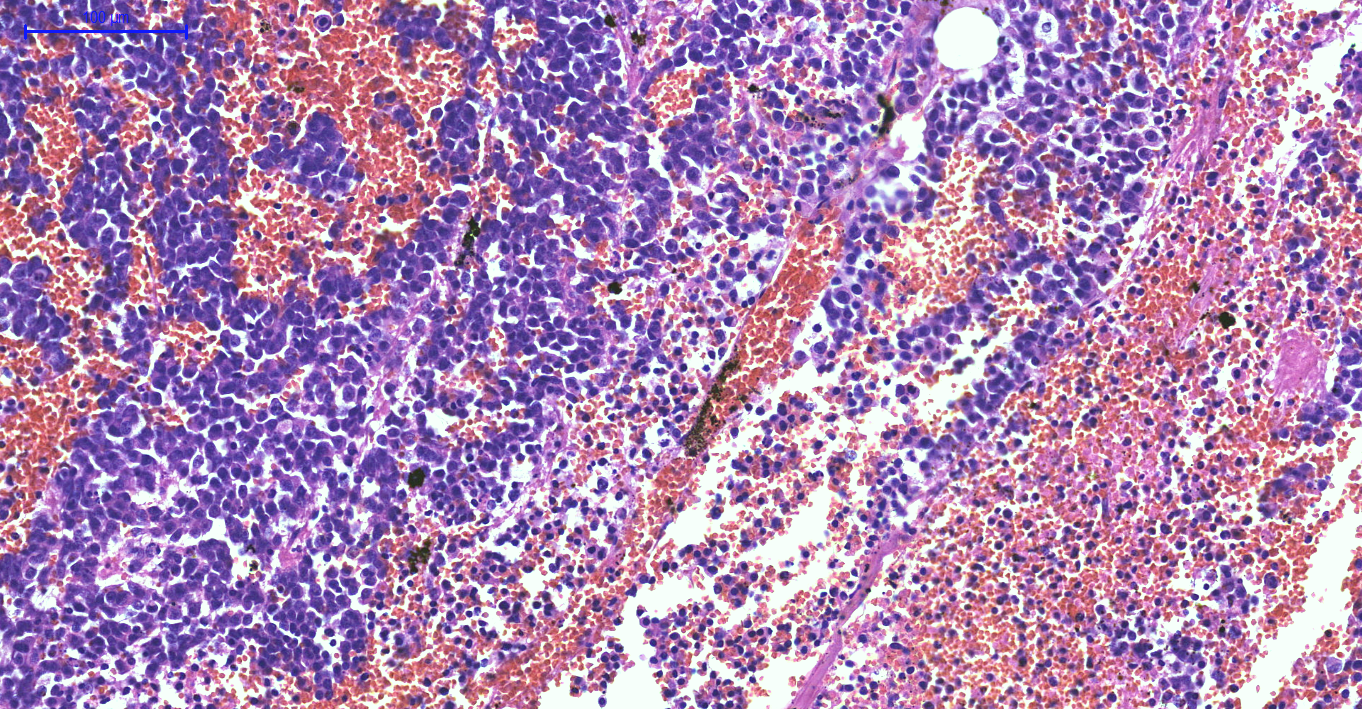

Supplement: Supplementary file 3 — Fig. 3c TW37–1 HE. (BMP 2830 kb) [file 12885_2019_5439_MOESM3_ESM.bmp]

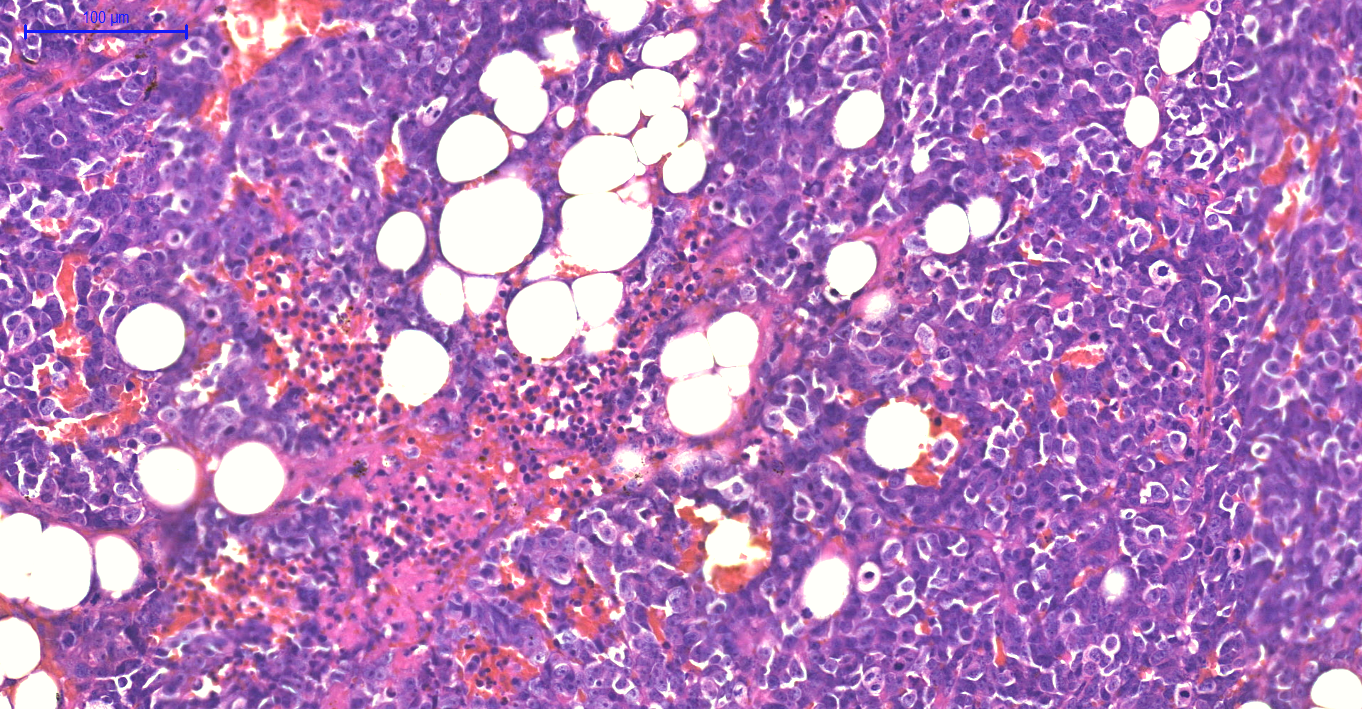

Supplement: Supplementary file 4 — Fig. 3c TW37–2 HE. (BMP 2830 kb) [file 12885_2019_5439_MOESM4_ESM.bmp]

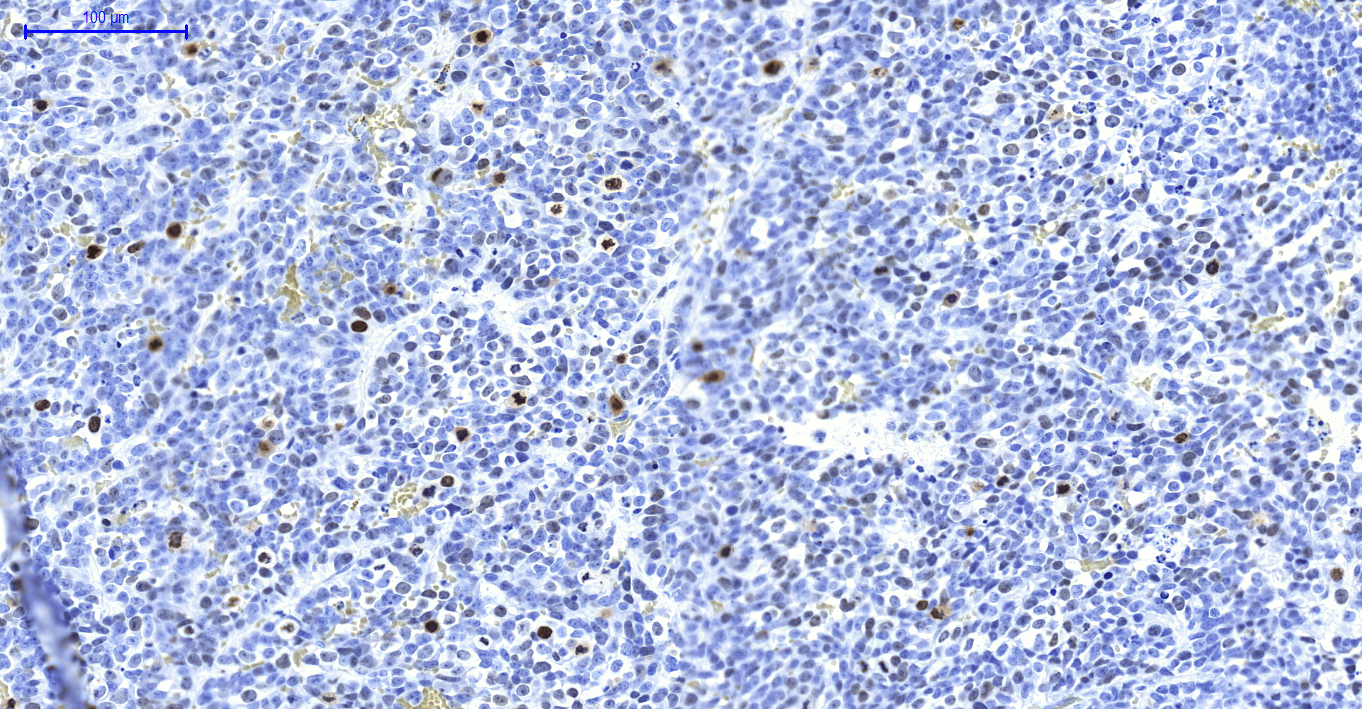

Supplement: Supplementary file 5 — Fig. 3c Control1 Ki-67. (BMP 2830 kb) [file 12885_2019_5439_MOESM5_ESM.bmp]

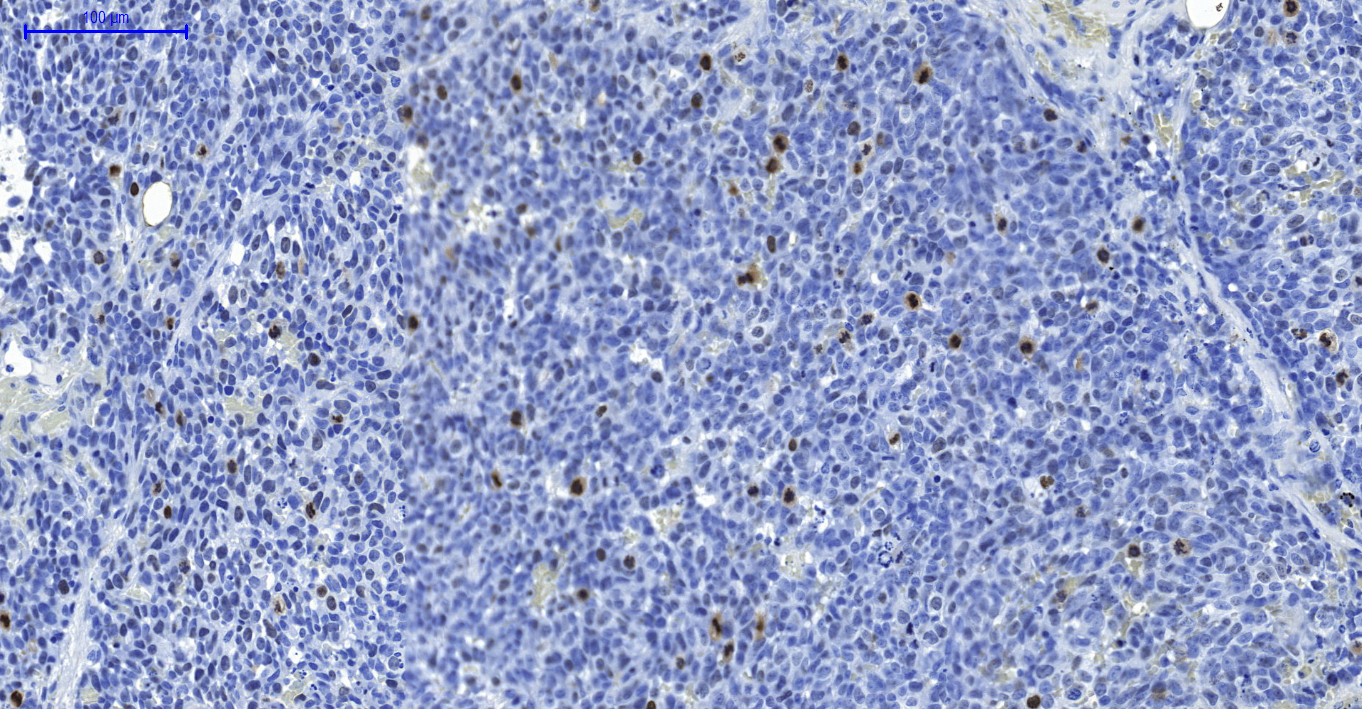

Supplement: Supplementary file 6 — Fig. 3c Control2 Ki-67. (BMP 2830 kb) [file 12885_2019_5439_MOESM6_ESM.bmp]

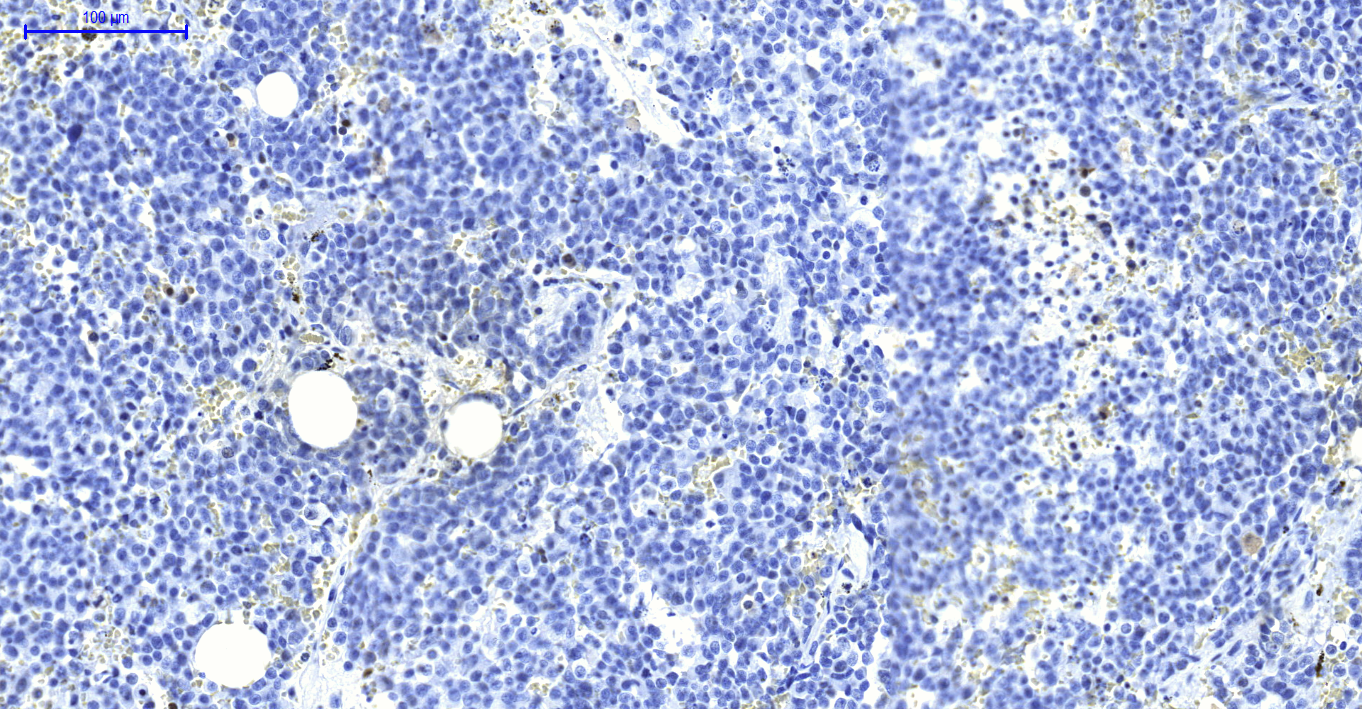

Supplement: Supplementary file 7 — Fig. 3c TW37–1 Ki-67. (BMP 2830 kb) [file 12885_2019_5439_MOESM7_ESM.bmp]

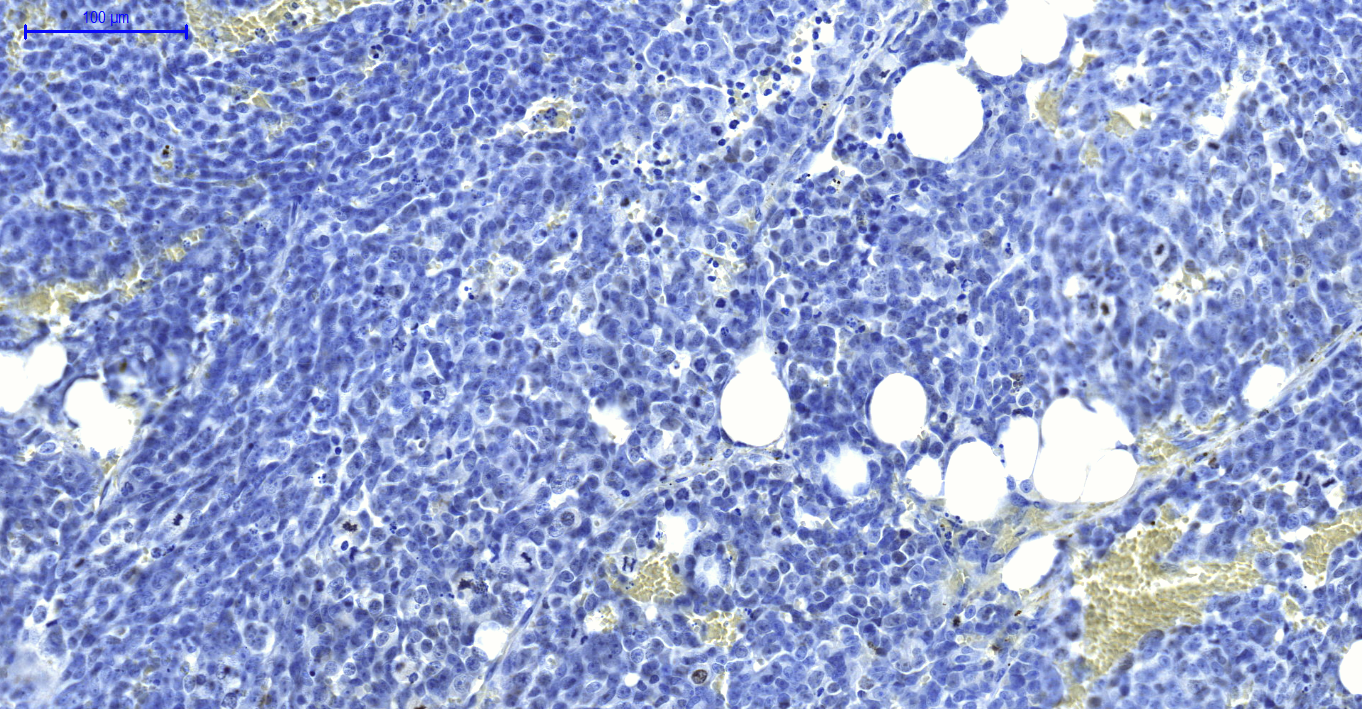

Supplement: Supplementary file 8 — Fig. 3c TW37–2 Ki-67. (BMP 2830 kb) [file 12885_2019_5439_MOESM8_ESM.bmp]

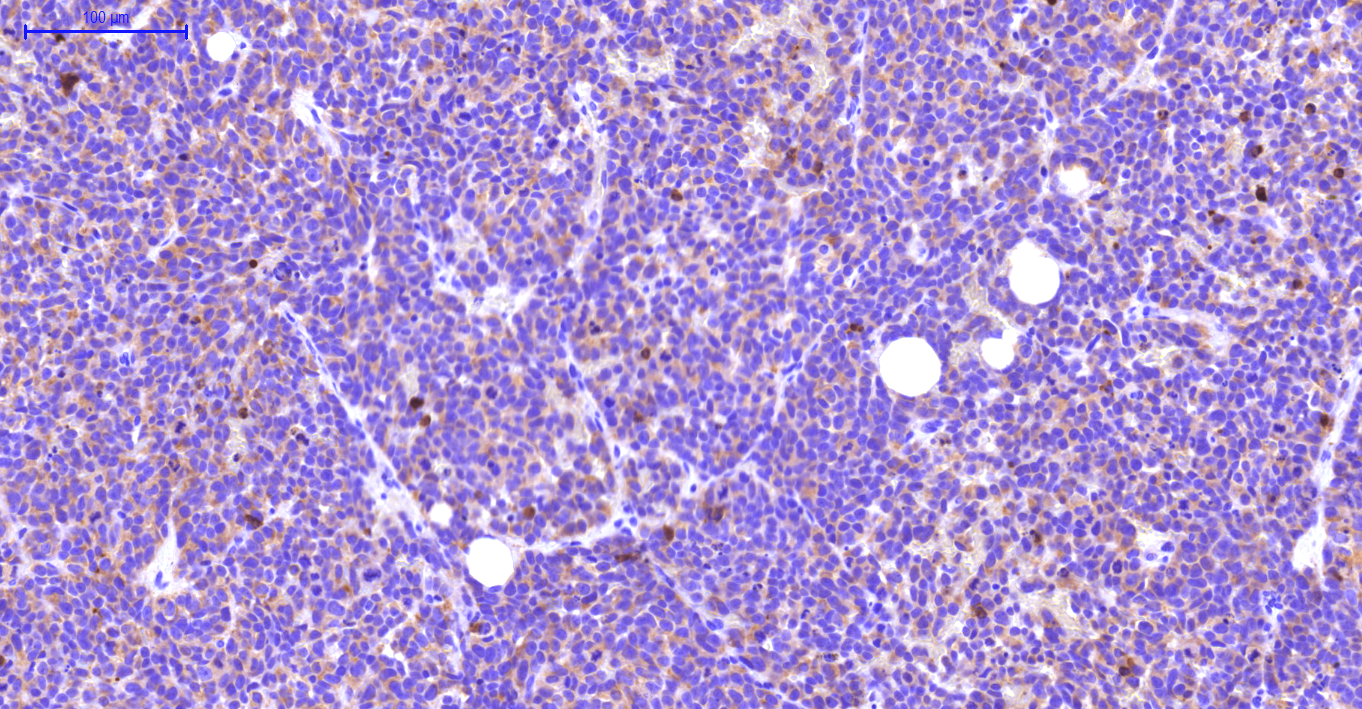

Supplement: Supplementary file 9 — Fig. 3c Control1 Casp3. (BMP 2830 kb) [file 12885_2019_5439_MOESM9_ESM.bmp]

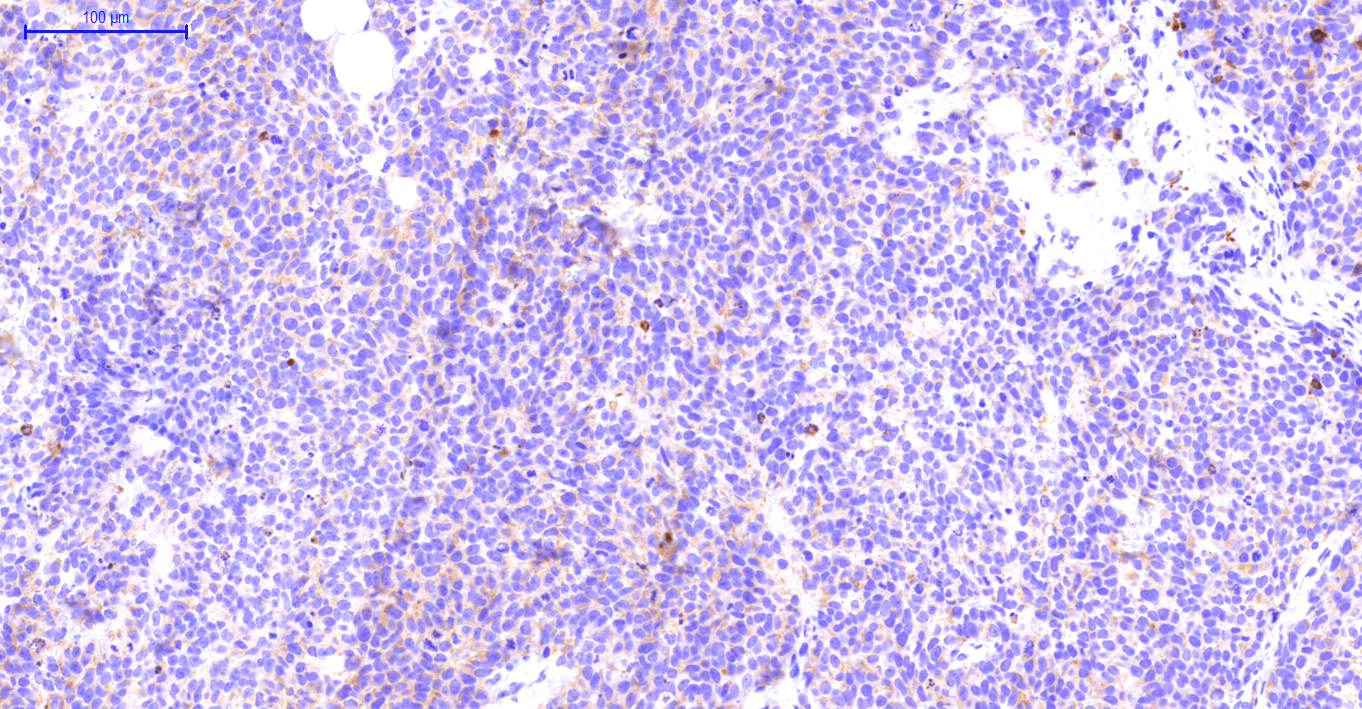

Supplement: Supplementary file 10 — Fig. 3c Control2 Casp3. (BMP 2830 kb) [file 12885_2019_5439_MOESM10_ESM.bmp]

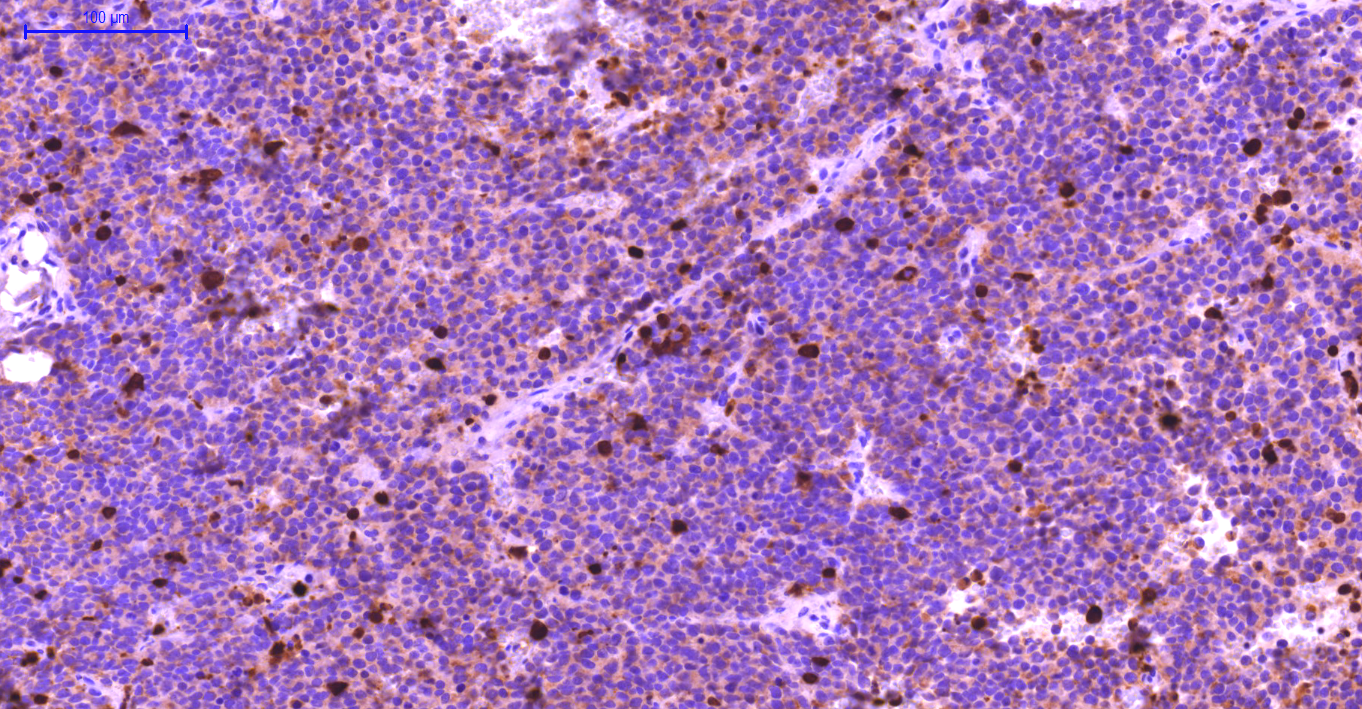

Supplement: Supplementary file 11 — Fig. 3c TW37–1 Casp3. (BMP 2830 kb) [file 12885_2019_5439_MOESM11_ESM.bmp]

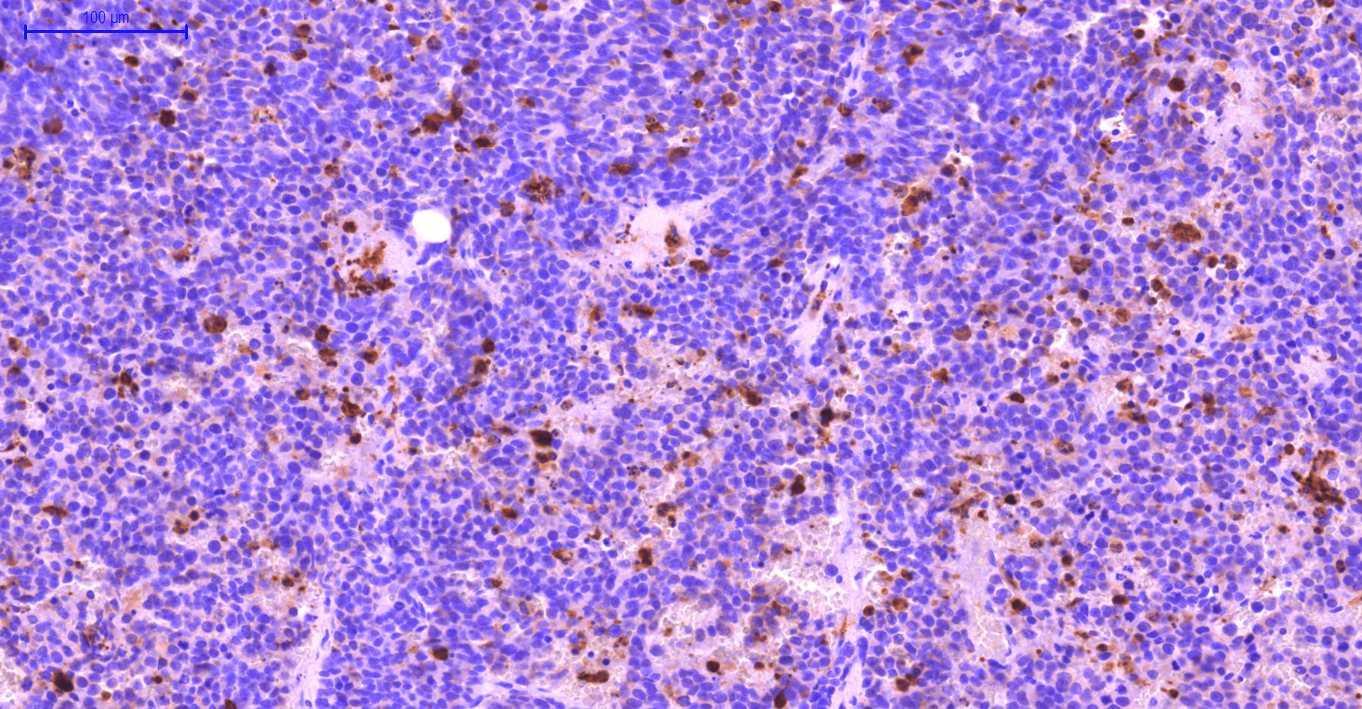

Supplement: Supplementary file 12 — Fig. 3c TW37–2 Casp3. (BMP 2830 kb) [file 12885_2019_5439_MOESM12_ESM.bmp]
